# Memristor-CMOS Hybrid Circuit for Temporal-Pooling of Sensory and Hippocampal Responses of Cortical Neurons

**DOI:** 10.3390/ma12060875

**Published:** 2019-03-15

**Authors:** Tien Van Nguyen, Khoa Van Pham, Kyeong-Sik Min

**Affiliations:** School of Electrical Engineering, Kookmin University, Seoul 02707, Korea; tiennv@kookmin.ac.kr (T.V.N.); khoapv@kookmin.ac.kr (K.V.P.)

**Keywords:** memristor-CMOS hybrid circuit, temporal pooling, sensory and hippocampal responses, cortical neurons, hierarchical temporal memory, neocortex

## Abstract

As a software framework, Hierarchical Temporal Memory (HTM) has been developed to perform the brain’s neocortical functions, such as spatial and temporal pooling. However, it should be realized with hardware not software not only to mimic the neocortical function but also to exploit its architectural benefit. To do so, we propose a new memristor-CMOS (Complementary Metal-Oxide-Semiconductor) hybrid circuit of temporal-pooling here, which is composed of the input-layer and output-layer neurons mimicking the neocortex. In the hybrid circuit, the input-layer neurons have the proximal and basal/distal dendrites to combine sensory information with the temporal/location information from the brain’s hippocampus. Using the same crossbar architecture, the output-layer neurons can perform a prediction by integrating the temporal information on the basal/distal dendrites. For training the proposed circuit, we used only simple Hebbian learning, not the complicated backpropagation algorithm. Due to the simple hardware of Hebbian learning, the proposed hybrid circuit can be very suitable to online learning. The proposed memristor-CMOS hybrid circuit has been verified by the circuit simulation using the real memristor model. The proposed circuit has been verified to predict both the ordinal and out-of-order sequences. In addition, the proposed circuit has been tested with the external noise and memristance variation.

## 1. Introduction

The neocortex occupying most of the brain’s surface area has been believed to perform the most human-like functions such as intelligence, cognition, etc. among all human organs. It is just 2.5-mm thick and is composed of six layers [1,2,3]. All six neocortical layers have the same columnar architecture, where the neocortical neurons are connected in both the vertical and horizontal directions to form various feedback and feedforward paths to communicate with each other. Anatomical experiments have observed the columnar architecture consistently through the entire neocortex [4,5]. This fact may hint that there is a canonical neural circuitry that can describe various neocortical functions with one model [6].

In this paper, we try to develop a memristor-CMOS hybrid circuit that can emulate the neocortex’s canonical neural circuitry by combining nanoscale memristor crossbars with CMOS peripheral circuits. Memristors have been studied intensively for many years for their possible use of neuromorphic hardware since the first experimental demonstration [7,8]. This is because the memristive behavior seems very similar with the biological synaptic plasticity, where the synaptic connection can be strengthened and weakened dynamically according to the sensory stimulus [9]. The ionic dynamics of memristors can also be used in implementing the reservoir computing hardware, where the cognitive function can be processed simply by applying the time-domain signals to the memristor-based reservoir [10]. Moreover, the memristor crossbars can be built in a 3-dimensional architecture by a CMOS-compatible fabrication process, where the 3-dimensionality is very similar to the anatomical view of the real biological neuron-synapse connections in the neocortex [11,12]. Also, the memristor crossbar can perform a bitwise parallel operation which has been thought as one of the key aspects of energy-efficient computing of the human brain’s cognition, compared to modern state-of-the-art computers [13,14].

**Figure 1:**
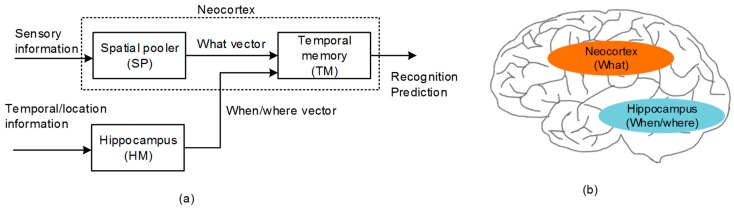
(**a**) The functional block diagram of Hierarchical Temporal Memory (HTM): The spatial pooler receives the sensory information from various sensory organs and forms the Sparse Distributed Representation (SDR) output representing the collective cortical neurons activated in response to the sensory information. The temporal memory learns the sequence of items that are represented by the SDR vectors by combining the sensory information with the temporal information. (**b**) The cross-sectional view of the human brain: Here, the neocortex and hippocampus regions are shown for processing the “what” and “when/where” information, respectively.

As a software framework for modeling the neocortical function, Hierarchical Temporal Memory (HTM) has been developed recently [15,16,17,18,19,20]. Figure 1a shows a functional block diagram of HTM that is composed of the Spatial Pooler (SP) and Temporal Memory (TM). SP receives the sensory information to learn the cortical representation. As a result, SP generates Sparse Distributed Representation (SDR) [16]. SDR is a mathematical description for representing the cortical neurons that may be activated or deactivated in response to the sensory information from the cochlea, retina, etc. Actually, SP was proposed as a software algorithm in the HTM software framework [15,16,17,18,19,20]. To realize the spatial pooling with hardware, we developed the spatial-pooling memristor crossbar circuit in a previous work, where the circuit could convert the sensory information to the SDR that meant the representation of cortical neurons [21].

The temporal memory (TM) in Figure 1a puts together the “what” and “when/where” vectors that come from the spatial pooler and hippocampus model, respectively [22,23]. By combining the “what” with “when/where”, the temporal memory can perform both the spatial recognition and the temporal prediction. For the temporal prediction, the representation of temporal succession (“when”) should be segregated from the representation of the content (“what”), as shown in Figure 1a [24,25]. From the experimental observations, the hippocampus has been known to play a central role in encoding the information of ordinal sequences (“when”) [25,26], whereas the representation of the content (“what”) has been known to come from the neocortex, as indicated in Figure 1b.

The representation of the temporal sequence (“when”) can be extended to the spatial sequence (“where”) [27,28]. For example, the order of the words during reading depends on where one is looking (“where”). However, the order of the words during listening can be interpreted as the temporal sequence (“when”’). Actually, every principal neuron in the hippocampus can work as either a “place cell” or “time cell” [29]. By doing so, the hippocampus can model both the temporal (“when”) and spatial (“where”) information with the same kinds of representation. Thus, we can think that the spatial sequence of location information is one case of the temporal sequence [26].

Though HTM has been developed as the software framework for performing the neocortex’s cognition, it should be realized with hardware not only to mimic the neocortex’s function but also to exploit its architectural benefit. One reason for this need of a hardware version is the demand of the edge-computing devices in the Internet of Things (IoT) era [30,31,32]. For the near-sensor processing and computing of IoT devices, the speed and power benefit due to the bitwise parallel-processing of memristor crossbars can be very important in terms of the possibility of real-time and on-chip cognitive functions for various edge-computing applications [32]. Thus, the neocortex’s cognitive function combined with the crossbar’s architectural merit can accelerate the transition from the HTM software framework to its hardware emulator [30].

To implement HTM by hardware not software, in this paper, we propose a new memristor-CMOS hybrid circuit for realizing the temporal-pooling function of the human brain, which is composed of the input and output layers, to mimic the temporal prediction of neocortical neurons. In the hybrid circuit, the input layer has proximal and basal/distal dendrites to combine the sensory information with the temporal/location information. The output layer composed of the same circuitry with the input layer can perform a prediction by integrating the temporal information through the basal/distal dendrites. In this paper, the input and output layers realized with the memristor-CMOS hybrid circuit are verified to perform the temporal recognition and prediction that are the same functions within the human brain’s neocortex.

## 2. Proposed Methods

Memristor crossbars are thought to be very suitable in mimicking the anatomical and functional architecture of neocortex. Memristive behaviors seem similar with the synaptic plasticity of biological neurons. Moreover, the 3-D connectivity of crossbars can be useful in realizing the real neuronal 3-D architecture of the neocortex. Also, the crossbars can perform a bitwise parallel computation, as the pyramidal neurons do in the neocortical layers. To develop the neocortex-mimicking memristor crossbar, first, we need to understand the functional model of neocortical columns and layers [33,34].

**Figure 2:**
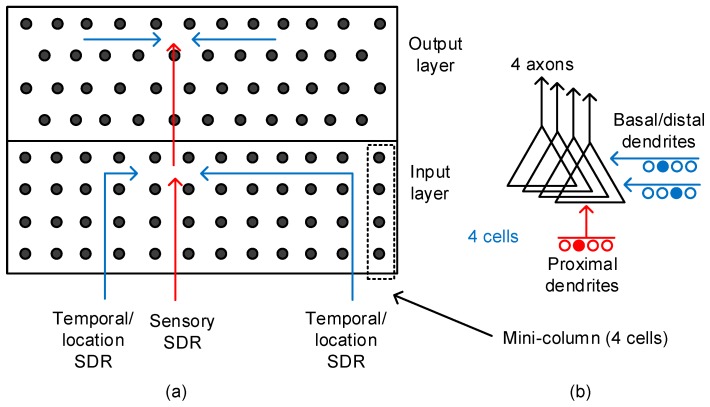
(**a**) The conceptual model of temporal memory architecture: The red and blue lines represent the proximal and basal/distal dendrites, respectively. (**b**) The schematic of a pyramidal neuron with a single proximal dendrite and multiple basal/distal ones. The number of output axons can be multiple too. Here, we showed 4 axons to constitute one mini-column with 4 cells. The pyramidal neurons are known as the majority of neocortical neurons.

Figure 2a shows the conceptual model of temporal memory composed of input-layer and output-layer neurons [23]. From previous experimental observations, the HTM theory deduced a couple of rules to describe the neocortex’s operation. First, it is assumed that the input-layer neurons receive the sensory information though the single proximal dendrite [23]. The synapses connected to this proximal dendrite are involved in only local signal-processing, as shown in Figure 2a. They do not communicate with the neurons outside the local region. The proximal dendrite is more likely to form short-distance vertical connections to receive the sensory information. On the contrary, the basal/distal dendrite is responsible for long-distance horizontal communication [23]. The dendrite can receive information from distantly located regions such as the hippocampus. One thing to note is that one neocortical neuron is allowed to have only single proximal dendrite. However, the basal/distal can have multiple.

Figure 2a also shows the output-layer neurons that are basically the same as the input-layer ones. The proximal dendrite is for short-and-direct connections from the input-layer neurons. The output-layer neurons can receive long-distance information horizontally through multiple basal/distal dendrites for the temporal integration of “when” vectors. The two-layer model is regarded as a general feature of the neocortex and can be used as an elemental unit in realizing the memristor-based temporal-pooling crossbar [23].

Figure 2b shows the schematic of a pyramidal neuron that incorporates the axonal and dendritic connections. The proximal dendrite receives the direct feed-forward inputs from the sensory organs. The basal/distal dendrite can be driven by the long-distance signals from far away regions, such as the hippocampus.

**Figure 3:**
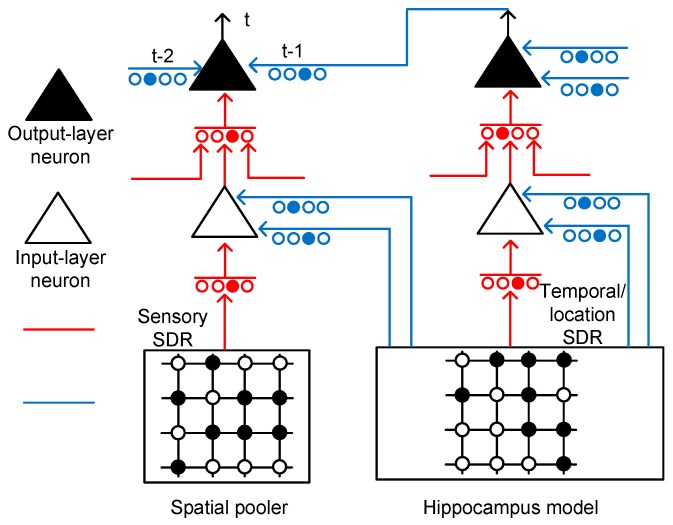
The conceptual schematic of a temporal-pooling memristor crossbar composed of input-layer and output-layer neurons: The input-layer neuron receives sensory SDR and temporal/location SDR from the spatial pooler and hippocampus model, respectively. The sensory and temporal/location SDR are generated from the spatial-pooling memristor crossbar that was developed in a previous work [21]. The output-layer neuron can perform a prediction by integrating the temporal information through multiple basal/distal dendrites.

Figure 3 shows the conceptual schematic of the temporal-pooling memristor crossbar composed of input-layer and output-layer neurons. The input-layer neurons receive sensory SDR and temporal/location SDR from the spatial pooler and hippocampus model, respectively. The sensory SDR vectors are connected with the proximal dendritic synapses. The basal/distal dendrites of the input-layer neurons receive hippocampal responses that contain the temporal and location information.

The output-layer neuron in Figure 3 has the same circuitry as the input-layer neuron, as shown in Figure 2b. The proximal connection of the output-layer neuron comes from the axonal output of an input-layer neuron. The basal/distal dendrite can make the output-layer neuron a predicted state by depolarizing its body. If the body is depolarized enough by the previous basal/distal dendritic inputs, it can fire spikes sooner than the other output-layer neurons, if they are not in the predicted state. If the output-layer neuron is not in the predicted state, it cannot fire spikes, even though it receives the same feedforward input as the predicted-state neuron. Only the predicted-state neuron which is depolarized already can fire spikes in response to the proximal dendritic input.

**Figure 4:**
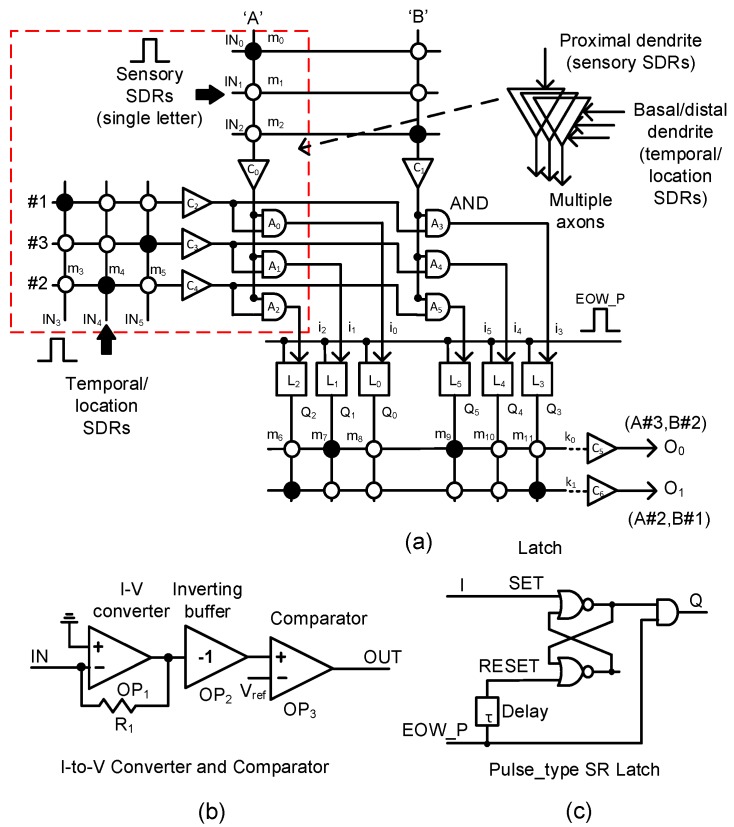
(**a**) The schematic of the proposed memristor-CMOS hybrid circuit for the temporal pooling of sequences such as words, sentences, etc: The input layer is composed of the memristor crossbars for sensory and temporal/location SDRs, the current-to-voltage converters, comparators, the AND gates, etc. The output layer is composed of the memristor crossbars, converters, comparators, latches, etc. (**b**) The schematic of the current–voltage converter and comparator and (**c**) the schematic of the pulse-type set-reset latch.

In Figure 4a, we propose a memristor-CMOS hybrid circuit that has the input and output layers for the temporal-pooling of sequences such as words, sentences, etc. Here, the sensory SDR vectors enter the proximal dendrites of m_0_, m_1_, m_2_, etc. The temporal/location SDR vectors are connected to the basal/distal dendrites of m_3_, m_4_, m_5_, etc. One thing to note in Figure 4a is that each neuron is allowed to have only a single proximal dendrite. However, for the basal/distal ones, the neuron can have multiple dendrites, as explained in Figure 2. The sensory SDR and temporal/location SDR are collectively received by the input-layer neurons. The column current of the sensory SDR “A” is delivered to C_0_, where the column current is converted to a voltage and then compared with the threshold. The detailed schematic of C_0_ is shown in Figure 4b. Similarly, the column current of “B”’ is delivered to C_1_. The temporal/location SDR vectors of “#1”, “#3”, and “#2” generate the row currents which are delivered to C_2_, C_3_, and C_4_, respectively. A_0_, A_1_, and A_2_ are the AND gates that combine the sensory information of “A”’ with the temporal/location SDRs of “#1”, “#3”, and “#2”, respectively. The outputs of A_0_, A_1_, and A_2_ are represented with i_0_, i_1_, and i_2_, respectively. They enter the pulse-type set-reset latches of L_0_, L_1_, and L_2_, respectively. The pulse-type set-reset latch is shown in Figure 4c. L_0_ can be set if the SDR “A” and SDR “#1” are recognized at the same time. L_1_ is set for “A” and “#3”. L_2_ is switched to the SET state for “A” and “#2”. Similarly, L_3_, L_4_, and L_5_ can respond to the input SDR of “B#1”, “B#3”, and “B#2”, respectively. The set-reset latch in Figure 4c is reset by the delayed version of the “EOW_P” pulse from the delay line τ. Here, “EOW_P” means the pulse indicating the end of the word. “EOW_P” is generated when the word ends.

As mentioned earlier, if the sensory SDR of letter “A” and the location SDR of “#3” are applied to the input-layer neuron, Q_1_ is activated. Similarly, when the sensory SDR of “B” and the location SDR of “#2” are recognized, Q_5_ becomes high. When “EOW_P” is activated, the two latches of L_1_ and L_5_ keep Q_1_ and Q_5_ high, respectively, until the reset. Assuming that the dendritic synapses of the output neuron O_0_ are already put in the predicted state with “A#3” and “B#2”, m_7_ and m_9_ are already programmed LRSs (Low Resistance States) as a result of crossbar training. Here, the solid and open circles represent LRS and HRS (High Resistance State), respectively. At end-of-word, if the row current of k_0_ is larger than the output-layer neuron’s threshold, O_0_ becomes high. Actually, we can think that the k_0_ current represents the integration of temporal responses to the sensory/location SDRs of “A#3” and “B#2” because “A#3” and “B#2’ were already recognized at the previous time. Similarly, if “A#2” and “B#1” are recognized one by one, the row current k_1_ becomes larger than the threshold and can activate O_1_.

**Figure 5:**
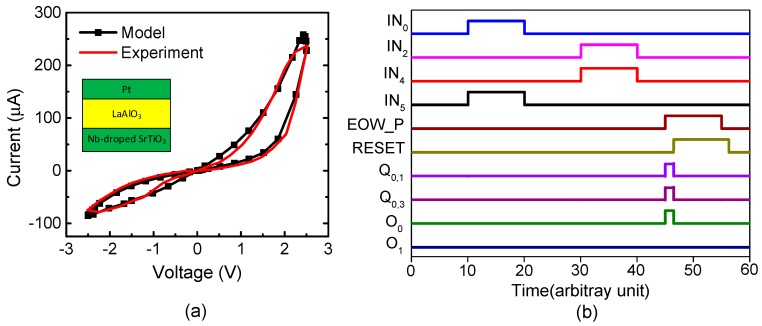
(**a**) The current–voltage relationships of memristors for the measurement and Verilog-A model: The black line represents the Verilog-A model of memristors used in the circuit simulation in this paper [35]. The red line is for the measurement [35]. The details of the measurement and the Verilog-A model were explained well in a previous publication [35]. (**b**) The waveforms of the proposed memristor-CMOS hybrid circuit for temporal pooling shown in Figure 4.

Figure 5a shows a current–voltage relationship of the measured memristor that was obtained by a Keithley-4200 (Semiconductor Characterization System, Tektronix, Inc., Beaverton, OR, USA) [35]. The measured memristor’s film is a Pt/LaAlO_3_/Nb-doped SrTiO_3_ stacked layer [35]. Here, the LRS and HRS were measured as 10 kΩ and 1 MΩ, respectively. The black line in Figure 5a represents the behavioral model of memristors [35]. The measured data are represented with the red line. The behavioral model described by Verilog-A was used in the circuit simulation of the hybrid circuits of memristors and CMOS in this paper. Here, the circuit simulation was performed using CADENCE SPECTRE (Cadence Design Systems, Inc., San Jose, CA, USA) and SAMSUNG 0.13-µm circuit simulation parameters [36]. The mathematical equations of the Verilog-A model of memristors were explained in a previous publication in detail [35].

Figure 5b shows the waveforms of Figure 4 obtained from the CADENCE (Cadence Design Systems, Inc., San Jose, CA, USA) circuit simulation with the memristor’s Verilog-A model in Figure 5a and the SAMSUNG 0.13-µm SPICE parameters. First, we assumed the sensory SDR of letter “A” and the location SDR of “#3” are generated by the spatial pooler. As a result, the IN_0_ and IN_5_ pulses are high, while the others are low in Figure 5b. By doing so, Q_1_ becomes high. Second, if the spatial pooler generates the sensory SDR of letter “B” and the location SDR “#2”, Q_5_ becomes high. At end-of-word, the pulse of “EOW_P” is enabled and the output neuron O_0_ becomes active. Here, the output neuron is already put in the predicted state by the previous signals of Q_1_ and Q_5_. After the output neuron O_0_ fires a pulse, O_0_ returns to low, as the typical integrate-and-fire neuron acts. To do so, the “EOW_P” pulse goes through the delay line τ and its delayed pulse resets the set-reset latches. The integrate-and-fire operation is realized very simply using the digital CMOS gates and the memristor crossbar, as shown in Figure 4a–c.

## 3. Results

**Figure 6:**
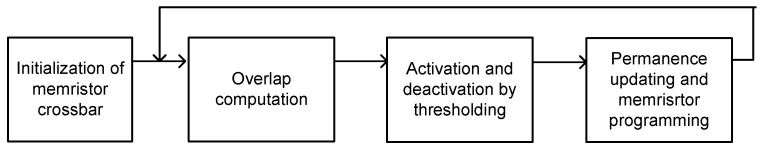
The operational steps of simple Hebbian learning of memristor crossbars: initialization, overlap computation, activation and deactivation by thresholding, and permanence updating and memristor programming. Here the memristor programming based on Hebbian learning does not need the complicated backpropagation calculation.

In this paper, we tested the proposed memristor-CMOS hybrid circuit of temporal pooling in Figure 4a with an EMNIST (Extension of Modified National Institute of Standards and Technology) data-set of handwritten letters [37]. For training the memristor crossbar to recognize EMNIST handwritten letters, we applied the simple Hebbian learning to 26 EMNIST letters from “a” to “z”. The operational steps of simple Hebbian learning of memristor crossbars is shown in Figure 6. Here, first, we initialized the memristor crossbar. Second, we calculated the amount of overlap between the input vector and the crossbar’s column or row. If the crossbar’s column or row has an overlap larger than the threshold, the column or row is activated. In this case, the permanence values of matched and unmatched memristor cells belonging to the activated column or row are increased and decreased according to the predetermined parameter Δ, respectively. If the permanence value becomes larger than 1 or less than 0, the memristor corresponding to the permanence is strengthened or weakened according to the memristor programing circuit. Here, we used the typical V_DD_/2 scheme for programming memristors. One thing to note is that the memristor programming based on Hebbian learning does not need the complicated backpropagation calculation [21]. By doing so, the proposed memristor-CMOS hybrid circuit can be very suitable to online learning because the hardware complexity of Hebbian learning is much simpler than that of a backpropagation-based system.

**Figure 7:**
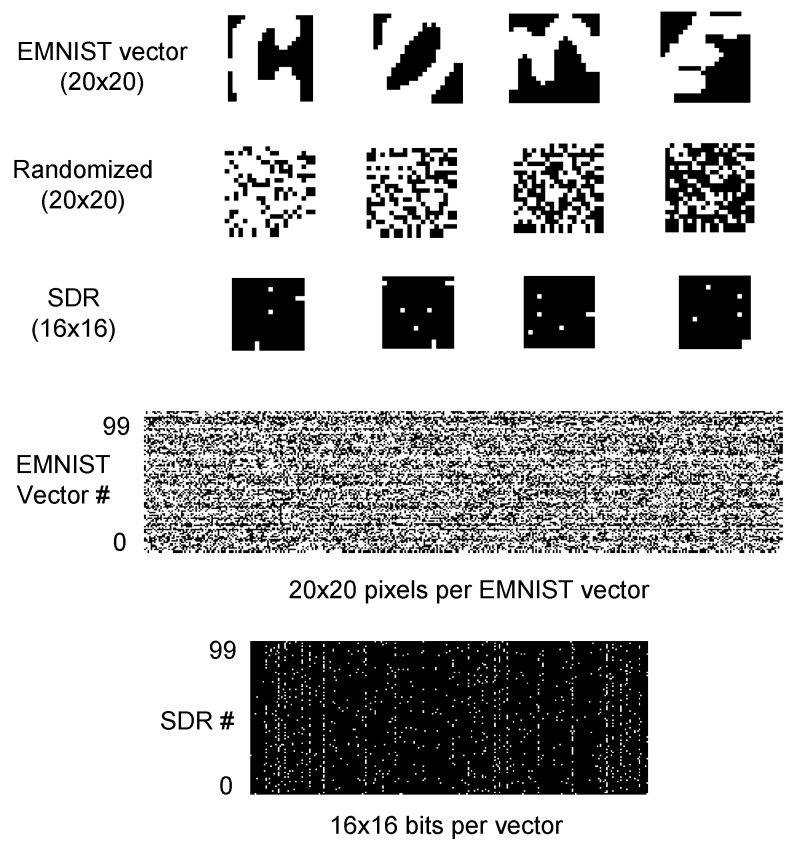
The first row shows the EMNIST handwritten letters of “c”, “o”, “m”, and “e”, respectively. The second row shows randomized images of EMIST handwritten letters. The third row are the SDRs that are obtained from the spatial-pooing memristor crossbar for the randomized images of “c”, “o”, “m”, and “e”. The fourth row shows 100 EMNIST input vectors. Each EMNIST vector is composed of 20 × 20 pixels. The fifth row shows 100 SDRs with 16 × 16 bits which are obtained from 100 EMNIST input vectors with 20 × 20 pixels. Among the 16 × 16 bits, only 2% of the bits become active to maintain the sparsity ratio around 2% by spatial-pooling for EMNIST vectors [21].

In this test, the 26 EMNIST letters have 60,000 training vectors. Each image is composed of 20 × 20 gray pixels. To estimate the recognition rate, we tested 10,000 execution vectors of an EMNIST letter. The first row in Figure 7 shows 4 images of EMNIST letters. They are “c”, “o”, “m”, and “e”, respectively. EMNIST vectors are randomized first and then applied to the spatial-pooling memristor crossbar proposed in a previous work [21]. The second row in Figure 7 shows the randomized version of the EMNIST vectors. It should be noted that the memristor-CMOS hybrid circuit does not need to use the complicated random number generation circuit. Once we decided the randomization function, we applied the same function to all the test vectors without changing it for every vector [21]. Thus, we did not use the random number generator circuit in a previous work [21].

If we perform the spatial pooing with 256 columns, we can obtain 16 × 16 SDRs from 20 × 20 EMNIST input vectors. The third row in Figure 7 shows the SDRs that are obtained from the spatial-pooing memristor crossbar for the randomized images of “c”, “o”, “m”, and “e”, respectively. The fourth row in Figure 7 shows the pixel map of 100 EMNIST test vectors. The average sparsity of the EMNIST test vectors is as high as 55.8%; that means 55.8% of the pixels can be white. On the contrary, the SDRs from the spatial-pooling crossbar have a sparsity as low as 2%. The fifth row shows 100 SDRs with 16 × 16 bits. Among the 16 × 16 bits of each SDR, only 2% of the bits become active by the spatial-pooling of the 20 × 20-pixel EMNIST vector. This low sparsity of SDRs is very useful in cognitive computations such as union, pattern matching, etc. [38]. In addition, the small number of active bits can reduce the number of LRS cells in a memristor crossbar. By doing so, the power consumption and sneak-leakage problem can be improved much in the spatial-pooling crossbar [39].

**Figure 8:**
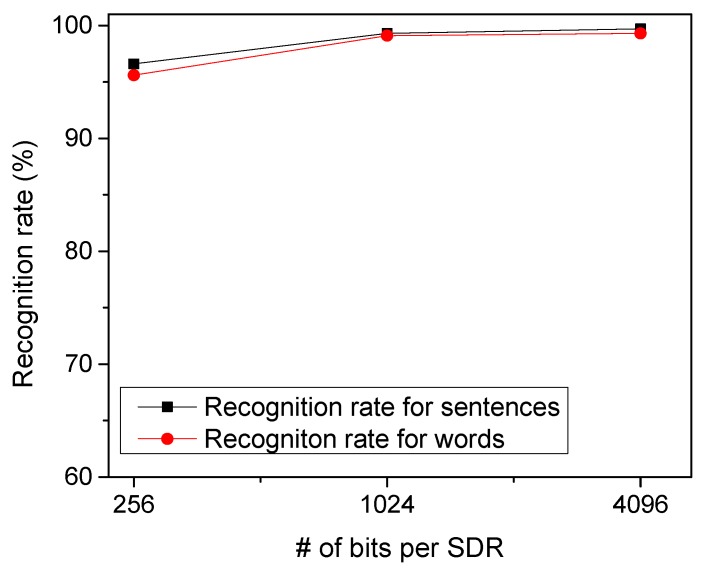
The recognition rate of words and sentences with varying the number of bits per SDR.

To test the temporal-pooling memristor-CMOS hybrid circuit in Figure 4a, we put together EMNIST handwritten letters to form arbitrary words. Figure 8 shows the recognition rate of the proposed temporal-pooling circuit for the 40 words tested in this paper. The recognition rate of words is estimated as high as 95.6%, 99.1%, and 99.3% for 256-bit SDRs, 1024-bit SDRs, and 4096-bit SDRs, respectively. One thing to note is that the recognition rate of words is much better than the recognition rate of EMNIST letters. This is because the temporal-pooling circuit interprets both sensory and temporal/location information together, as indicated in Figure 2. Combining the sensory SDRs with the location SDRs makes the recognition of words better than the recognition of letters. Figure 8 also shows the recognition rate of sentences as high as the rate of words. The recognition rate of sentences is simulated 96.5%, 99.3%, and 99.7% for 256-bit SDRs, 1024-bit SDRs, and 4096-bit SDRs, respectively. Here, the number of sentences tested in Figure 8 is 10.

**Figure 9:**
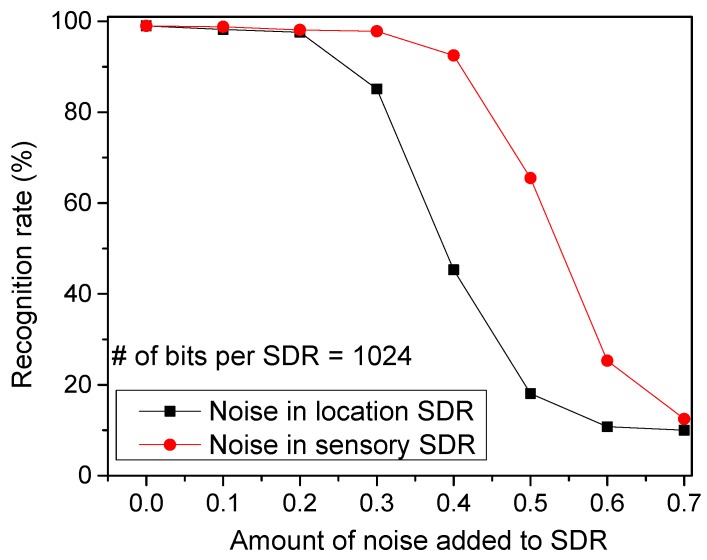
The recognition rate of words by varying the amount of noise added to location SDRs and sensory SDRs: The red circles represent the recognition rate for the noise added to the sensory SDRs. The black boxes are for the noise added to the location SDRs.

Figure 9 shows the recognition rate by varying the amount of noise added to SDRs. The noise is added by randomly flipping a fraction of the active bits to inactive, and vice versa so that the sparsity can be maintained constant. As shown in Figure 4a, the temporal-pooling circuit receives both the sensory and location SDRs from the spatial pooler. Here, the red circles represent the recognition rate for the noise added to the sensory SDRs. The black boxes are for the noise added to the location SDRs. From this figure, the noise added to the location SDRs seems more critical in terms of recognition rate. If a noise as large as 40% is added to the location SDRs, the recognition rate becomes as low as 45.3%. However, the rate can be as high as 92.5% for the same amount of noise added to the sensory SDRs.

**Figure 10:**
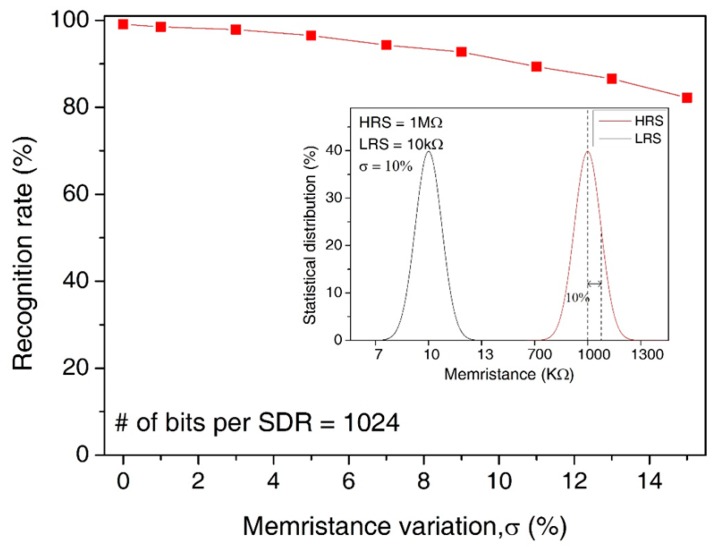
The recognition rate of words by increasing the percentage variation in memristance from 0% to 15%: The inset figure shows the statistical distribution of LRS and HRS for the memristance variation = 10%.

In Figure 10, we assumed the statistical distribution of LRS and HRS with the memristance variation = 10%, as shown in the inset figure. Here, the main figure shows the recognition rate by varying the amount variation in memristance from 0% to 15%. Here, the median values of HRS and LRS are assumed as 1 MΩ and 10 KΩ, respectively. Though the variation is as large as 15%, the recognition rate is still as high as 85.9%. The loss of recognition rate for the variation = 15% is only as small as 13.2% compared to the variation = 0%.

**Figure 11:**
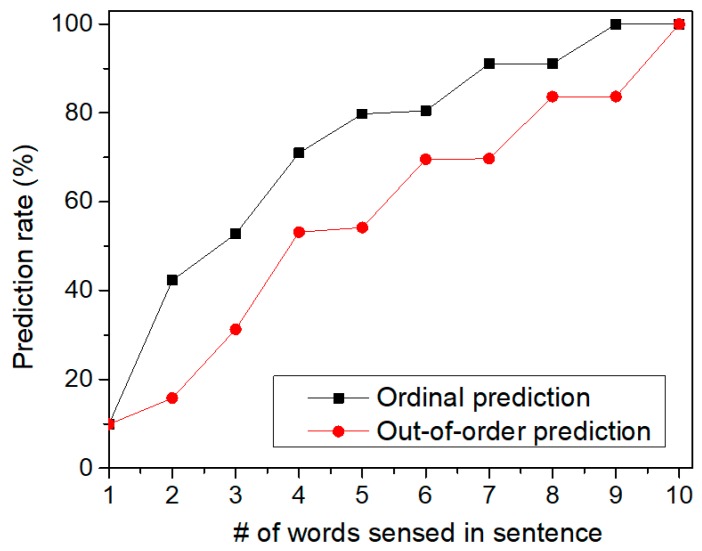
The prediction rate of sentences by increasing the number of words sensed for recognizing the sentences: Here, both the ordinal and out-of-order sequences can be recognized by the temporal-pooling memristor crossbar circuit proposed in this paper.

Figure 11 shows the prediction rate of sentences by increasing the number of words sensed in the tested sentence. Here, we tested two cases of sequences which are ordinal and out-of-order sequences, respectively. First, let us explain the ordinal sequence. Assume that we try to recognize two sequences of “‘A-B-C-D-E” and “A-B-C-E-D”. Here, the first three SDRs are “A-B-C” which are the same for both sequences. Also, the fourth and fifth SDRs are different each other. If the first SDR of “A” comes to the memristor crossbar, it cannot distinguish the two sequences. Similarly, for the second SDR of “B”, the crossbar circuit also cannot make a judgement between “A-B-C-D-E” and “A-B-C-E-D”. However, if the fourth SDR is given to the crossbar, it can predict if the fifth SDR will be “E” or “D” according to the fourth SDR information. This is called the ordinal prediction in Figure 11, where the temporal-pooling circuit can predict the ordinal sequence of SDRs. Figure 11 shows the prediction rate of ordinal sentences. The prediction rate starts from zero. This means the crossbar can predict nothing at the starting time of prediction. If the first SDR is given, the crossbar starts to predict the rest words of the tested sentence. As the crossbar receives more words from the spatial pooler, the prediction becomes more accurate, as shown in Figure 11. When the crossbar receives the final SDR at the end of sentence (period symbol), the prediction rate in Figure 11 becomes equal to the recognition rate of sentences in Figure 8.

We also tested the prediction for out-of-order sequences in Figure 11. In the out-of-order prediction, the sequence of SDRs are out of order. In spite of the out-of-order sequence, the crossbar can accumulate the information of the sensed words over time. By doing so, the temporal-pooling circuit can guess what word should come next. This out-of-order prediction is exactly the same case as the crossword puzzle problem. In solving the crossword puzzle, we predict the word by accumulating the information of letters in the out-of-order sequence over time. When the temporal-pooling circuit is given only half words in the tested sentence, Figure 11 indicates that the crossbar can predict the ordinal and out-of-order sentences as accurate as 79.8% and 54.2%, respectively.

## 4. Discussion

In this section, we compare the proposed memristor-CMOS hybrid circuit with the previous sequential memristor crossbar [40] in terms of the memristor crossbar area, power consumption, and prediction of the ordinal and out-of-order sequences. The previous sequential memristor crossbar was designed not to consider the concept of location SDR in the crossbar, unlike the proposed temporal-pooling hybrid circuit in this paper [40]. Thus, the previous sequential scheme can recognize only the ordinal not the out-of-order sequence [40]. This is a very big disadvantage of the previous sequential scheme. For the power consumption, we had to program memristor cells of the serial chain one by one in the previous sequential crossbar to recognize the ordinal sequences [40]. This results in a large amount of programming power consumption in the previous scheme. On the contrary, the proposed temporal-pooling hybrid circuit does not demand the memristor programming in recognizing both the ordinal and out-of-order sequences. By doing so, the power consumption of the proposed temporal-pooling circuit can be almost as small as 1/29 of the previous scheme, as indicated in Table 1. One more thing to note is the CMOS peripheral circuit in Figure 4a consumes only a negligible amount of the power than the memristor crossbar. Actually, most of the power is consumed in the LRS cells in the crossbar. Thus, minimizing the number of LRS cells in the memristor crossbar is very critical not only for alleviating the sneak leakage problem but also for reducing the power consumption [21]. Comparing the memristor crossbar’s area between the previous and proposed schemes indicates the number of memristors of the proposed temporal-pooling hybrid circuit is estimated almost the same with that of the previous scheme, as shown in Table 1.

Finally, we discuss here the practical applications of Hebbian-based HTM algorithm. Actually, if we compare the Hebbian-based HTM algorithm with the previous deep-learning ones such Convolutional Neural Networks, etc. for recognizing the benchmark image data-set, the deep learning outperforms the Hebbian-based HTM [21]. However, according to Numenta Inc. that developed HTM algorithm, the biologically inspired HTM can work best with data that meets the following characteristics: streaming data rather than batch data files, data with time-based patterns, many individual data sources where hand crafting separate models is impractical, subtle patterns that cannot always be seen by humans, and data for which simple techniques such as thresholds yield substantial false positives and false negatives [41]. This means that the Hebbian-based HTM algorithm can be more suitable to the area of Human-like sensory information such as the streaming data composed of anomaly patterns, as we showed in the case of the out-of-order prediction in Figure 11. On the contrary, for a static image data-set such as MNIST, CIFAR, etc., the conventional deep learning techniques can be better than HTM [21]. The real practical applications of the Hebbian-based HTM algorithm were explained in detail in previous publications [41,42]. In addition, the experimental results of memristor crossbars with Hebbian learning were shown in previous publications [43,44], where memristor’s conductance was trained by the Hebbian algorithm for various neuromorphic applications.

## 5. Conclusions

As a software framework, Hierarchical Temporal Memory (HTM) has been developed to perform the brain’s neocortical functions such as spatial and temporal pooling in software. However, it should be realized with hardware not software not only to mimic the neocortex’s function but also to exploit its architectural benefit. To do so, in this paper, we proposed the memristor-CMOS hybrid circuit to realize the temporal-pooling function of human brain, which is composed of the input and output layers to mimic the neocortical neurons. In the hybrid circuit, the input layer has proximal and basal/distal dendrites to combine the sensory information with the temporal/location information caused from the brain’s hippocampus. Using the same crossbar architecture, the output layer can perform predictions by integrating the temporal information through the basal/distal dendrites. For training the memristor-CMOS hybrid circuit, we used only simple Hebbian learning, not the complicated backpropagation algorithm. Due to the simple hardware of Hebbian learning, the hybrid circuit can be thought very suitable to online learning.

The proposed memristor HTM circuit was verified by the circuit simulation using memristor’s Verilog-A model obtained from the measurement. The proposed crossbar circuit was tested to recognize words and sentences that are composed of EMNIST data-set of handwritten letters. The recognition rate for sentences was estimated as high as 96.5% for 256-bit Sparse Distributed Representation (SDR). In addition, the proposed circuit was tested with the external noise and memristance variation. The proposed temporal-pooling circuit also was verified to perform both the ordinal and out-of-order predictions. When the proposed circuit was given only half words in the tested sentence, it could predict the ordinal and out-of-order sequences with the accuracy of 79.8% and 54.2%, respectively.

## Figures and Tables

**Table 1:** A comparison of the memristor crossbar area, power consumption, and prediction of the ordinal and out-of-order sequences.

Scheme	The Previous Sequential Memristor Crossbar [40]	The Proposed Memristor-CMOS Hybrid Circuit of Temporal Pooling
The number of memristors (Memristor crossbar area)	17556	17027
The amount of power consumption (LRS = 1 MΩ, HRS = 100 MΩ)	151.5 µW	5.24 µW
Prediction of ordinal sequences	O	O
Prediction of out-of-order sequences	X	O
